# Diagnosis of carbon monoxide exposure in clinical research and practice: A scoping review

**DOI:** 10.1371/journal.pone.0300989

**Published:** 2025-02-05

**Authors:** Phil Moss, Natasha Matthews, Rosalie McDonald, Heather Jarman

**Affiliations:** 1 St Georges’ Emergency Department Clinical Research Group, Emergency Department, St Georges’ Hospital, London, United Kingdom; 2 Population Health Research Institute, St George’s University of London, London, United Kingdom; University of Catania, ITALY

## Abstract

**Objective:**

To undertake a scoping review to identify methods and diagnostic levels used in determining unintentional, non-fire related carbon monoxide exposure.

**Design:**

Online databases and grey literature were searched from 1946 to 2023 identifying 80 papers where carbon monoxide levels were reported.

**Results:**

80 papers were included; 71 research studies and 9 clinical guidelines. Four methods were described: blood carboxyhaemoglobin (arterial or venous blood analysis), carbon monoxide oximetry (SpO2), expired carbon monoxide, and ambient carbon monoxide sampling. Blood analysis methods predominated (60.0% of the papers). Multiple methods of measurement were used in 26 (32.5%) of the papers. Diagnostic levels for carboxyhaemoglobin were described in 54 (67.5%) papers, ranging between 2% and 15%. 26 (32.5%) papers reported diagnostic levels that were adjusted for the smoking status of the patient.

**Conclusions:**

Four methods were found for use in different settings. Variability in diagnostic thresholds impairs diagnostic accuracy. Agreement on standardised diagnostic levels is required to enable consistent diagnosis of unintentional, non-fire related carbon monoxide exposure.

## Introduction

### Rationale

Carbon monoxide (CO) is a colourless, odourless gas produced by incomplete combustion of carbon compounds. It is reported as a significant cause of death from poisoning worldwide, leading to a population burden of 137 cases and 4.6 deaths per million in 2017 [1]. In the US data from the National Centre for Health Statistics show a reduction in death between 2015 and 2021 from 1,253 to 1,067 [2]. Exposure to CO leads to the formation of carboxyhaemoglobin (COHb) within the red blood cells, with CO binding to haemoglobin with an affinity 220 times that of oxygen. This results in a combination of tissue hypoxia from reduced oxygen delivery to the tissues and the generation of free radicals from CO-mediated damage to the cells [3–5]. Physiological breakdown of haem molecules produces endogenous CO [6]. Smokers have higher levels of endogenous CO compared to non-smokers [7]. This is why non-harmful levels of COHb are not set at zero. Exposure to CO can be both intentional and unintentional (accidental). Intentional exposure results from self-poisoning, usually through suicide using car exhaust fumes. Unintentional exposure can be further subdivided into fire-related exposure where there is a clear history of CO release, and non-fire related occurring due to exposure from outdoor or indoor sources. Outdoors it results from air pollution when CO is produced by vehicles and industry pollutants. Indoors the most common sources of CO are found in the home from incorrectly installed, poorly maintained, inappropriately used, or poorly ventilated fossil fuel and wood-burning heating and cooking appliances [8–10]. Threshold levels that cause harm are set by the World Health Organisation. Harmful exposure is defined by CO concentrations in parts per million (ppm) and duration of exposure i.e., 100 ppm for 15 minutes,35 ppm for 1 hour and 10 ppm for 8 hours [11]. An increase in CO-related morbidity and mortality has also been found after natural disasters where power outages lead to the inappropriate placement of portable generators and the use of malfunctioning propane heaters in the home [12–14].

Diagnosis of CO exposure in clinical practice is reliant on a clinical diagnostic triad: 1. symptoms consistent with CO exposure; 2. a recent history of CO exposure; and 3. elevated levels of the biomarker carboxyhaemoglobin (COHb) [15, 16]. CO exposure can have different clinical effects depending on the severity, although in low-level exposure, patients may not be aware of the exposure and COHb levels may not be raised. Diagnosis based on symptoms alone is challenging due to the non-specific nature of the presentation. Neurological symptoms predominate, particularly headache, with other symptoms such as dizziness, chest pain, and drowsiness [15, 16]. In the paediatric population CO can present with seizures, vomiting, headache, and nausea [17]. Exposure to elevated levels, or ongoing exposure to low levels of CO, can lead to long-term cognitive issues with impaired memory loss and motor deficit [18–20].

### Objectives

This scoping review aims to identify the current methods and diagnostic threshold levels used for determining exposure in unintentional, non-fire related (UNFR) CO exposure in clinical practice guidelines and research literature.

## Methods

This review follows the five-stage process described by Arksey and O’Malley for conducting scoping reviews and the Preferred Reporting Items for Systematic reviews and Meta-Analyses extension for Scoping Reviews (PRISMA-ScR) [21, 22].

### Stage 1—Identifying the research question

A preliminary exploration of the literature revealed differences in how CO is measured in practice, and in the diagnostic levels used to determine what constitutes harmful exposure. The research question for this review was: What are the current methods used in clinical practice guidelines and research to diagnose unintentional, non-fire related carbon monoxide exposure?

### Stage 2 –identifying relevant studies / search strategy

#### Search strategy

The search strategy was developed based on the expertise of the research team and a health information scientist and was used to generate the initial literature search. The search strategy was tested on a single database before being refined and replicated on other databases [S1 Table]. Searches were conducted using the MEDLINE, Cumulative Index to Nursing and Allied Health Literature [CINAHL] and Excerpta Medica [EMBASE] databases and performed by the health information scientist. Search terms were modified for each of the databases. This search was supplemented by grey literature identified through the TRIP Medical Database, NICE and TOXBASE to include published clinical guidance on CO diagnosis. References lists and citations of included studies were reviewed to identify additional articles meeting the inclusion criteria.

For search strategy see S1 Table.

#### Eligibility criteria

The inclusion criteria were defined as articles reporting CO exposure meeting the following characteristics:

- Original articles or conference abstracts published between 01/1946 and 08/2023
- Clinical guidelines published since 2000.
- Inclusion of at least one method of measurement of CO

The following exclusion criteria were applied:

- Articles where the source of exposure was intentional CO exposure or fire (where there are elevated levels of CO)
- Clinical review articles
- Written in language other than English.
- Articles not relating to human subjects.
- Articles relating to CO from tobacco smoking or outdoor air pollution.

#### Selection of studies for inclusion

The study selection criteria were established during the initial review process. Search results were imported into the Rayyan web application for sorting and screening [23]. Two reviewers [xx] and [xx] independently screened titles and abstracts against the inclusion criteria. Where there was not enough information to decide on inclusion using the abstract a full text review was carried out. Disagreements between reviewers were resolved through screening or discussion with a third reviewer [xx]. Further exclusions were made at full text review following agreement from all reviewers. Rationale for excluding articles were recorded.

### Stage 4 –charting the data

A data extraction form was developed, tested, and refined in Excel (Microsoft Office 365 E3, Redmond, Washington, US) to systematically list key components of the included articles. Three overarching categories were extracted: 1. Article characteristics (country, population, number of participants, study setting and design where relevant); 2. Method of measuring CO; and 3. Diagnostic levels for determining an abnormal COHb. A single reviewer extracted all the data from the articles and the data extraction form was reviewed by the other reviewers. Any discrepancies were resolved by discussion. Following guidance on conducting a scoping review, no quality appraisal of the included papers was performed [21]. See S2 Table.

### Stage 5 –assembling, summarising, and reporting the results

The identified studies were summarised, and relevant data was extracted. Content analysis was used to identify concepts within the papers. Data was presented narratively under the overarching categories. Geographical region, study design, methods for measurement of CO (categorised by type), and diagnostic levels.

## Results

**Fig 1:**
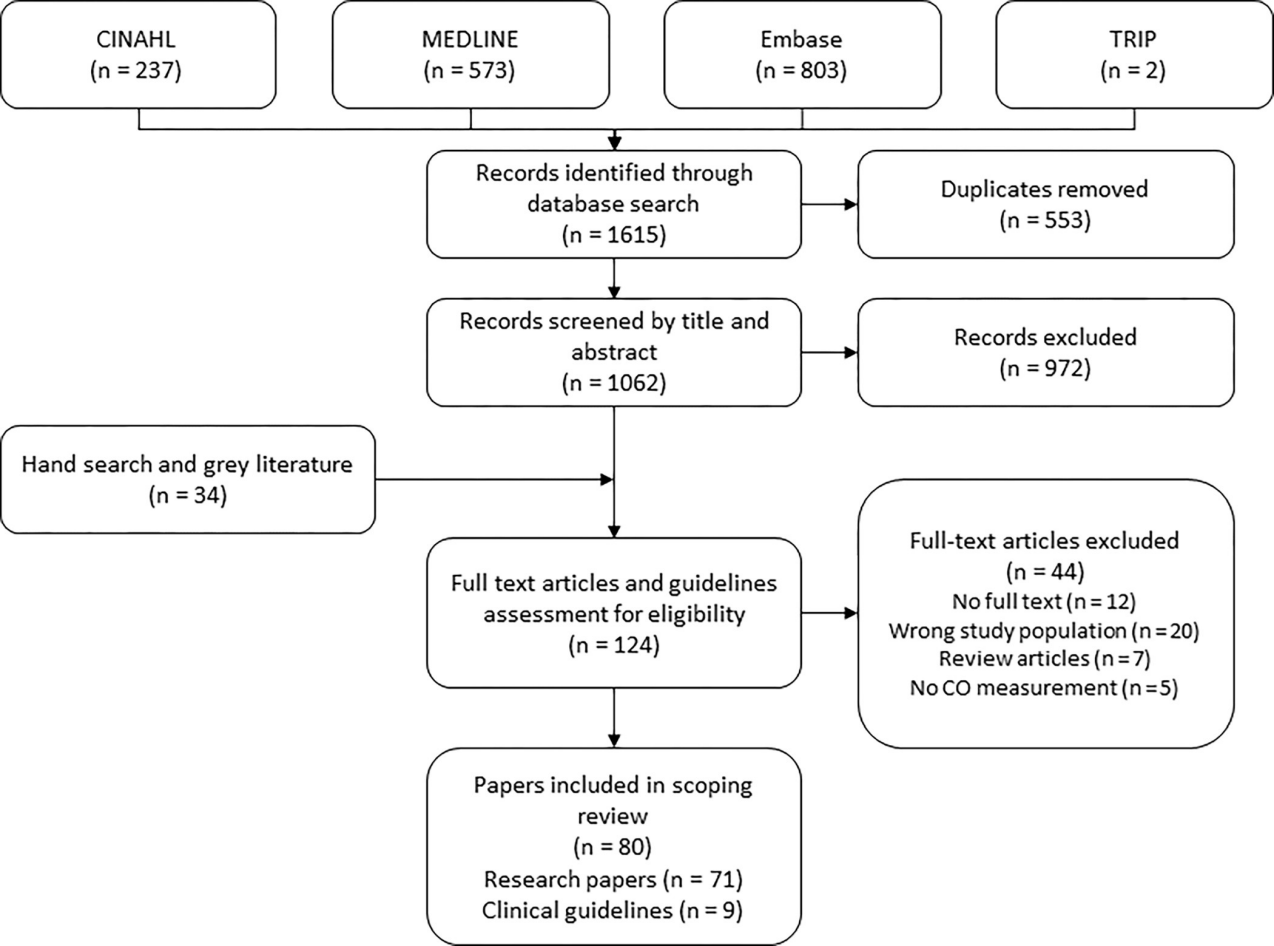
PRISMA flow chart.

Of 1615 articles, 80 articles were included. The most common reason for exclusion was the wrong population being studied including papers with fatalities as subjects, papers reporting correlations between symptoms and CO exposure, patients undergoing treatment for known exposure and papers using healthy volunteers as subjects. Fig 1 shows the PRISMA (Preferred Reporting Items for Systematic Reviews and Meta-Analyses) flow diagram detailing the results of the search and selection process.

### Paper characteristics

The characteristics of papers, including country, paper type, study design, setting for measurement of study sample, participant age (adult/child), number of participants, known or unknown exposure, source of exposure, method of measurement and diagnostic levels by smoking status and data collection method were inputted into data extraction tables (S3A and S3B Table). Overall, 32 counties published research or guidance, most were published in North America (n = 29, 35.8%). Papers identified were published between 1987 and 2023, 78.8% of which were published between 2007 and 2023. Nine clinical guidelines were identified (11.3%), [11, 16, 24–30] the remaining being research papers. Further categorisation by study methodology found observational studies predominated (65/71; 91.5%). Of these 38/71 (53.5%) were cross-sectional [17, 31–67], 17/71 (23.9%) case reports or case series [68–83], 7/71 (9.9%) cohort design [84–91]. The remaining (3/71; 4.2%) were diagnostic accuracy [92–94] Interventional studies numbered 6; 8.5% [95–100] In research papers the most frequent study setting for measurement was the emergency department (45.0%, n = 36/71) [17, 32, 34, 35, 38–41, 45, 47, 49, 51, 57–64, 70–72, 75–77, 79–82, 87, 88, 91, 93, 94]. The number of study participants ranged from 1 to 74880, with a median of 999 (IQR 34–771).

### Method of CO measurement

Across all papers, four methods for measurement of CO levels were identified (Table 1).

**Table 1:** Types of CO measurement methods identified in papers.

CO measurement method	Description
Blood carboxyhaemoglobin (COHb)	Spectrophotometric measurement detects derivatives of haemoglobin by using discrete narrow bands of electromagnetic waves to differentiate the degree of light absorption by each derivative [101].
CO oximetry (SpCO)	An external skin probe using multi-wavelength photometry to detect the presence of carboxyhaemoglobin (COHb) [93]. A strong correlation between SpCO and blood COHb is shown with Bland–Altman analyses [51, 59, 60, 92–94].
CO breath analysis (end-tidal exhaled CO)	End-expiratory breath measurement in parts per million (ppm) using an electrochemical sensor. COHb% can be calculated from the ppm value and closely correlates to blood COHb concentration [102].
Environment particulates (ambient CO measurement)	CO is measured using electrochemical sensors, typically a metal oxide semiconductor [103].

The majority of studies (40/71; 56.3%) and all but one of the guidelines (8/9; 88.9%) used venous blood to measure COHb levels [11, 16, 24–30, 32, 33, 35, 39–41, 43, 45, 47, 49, 51, 57–64, 68, 70–72, 74, 76–83, 85, 87, 88, 91–94, 96]. Second most reported across all paper types was SpCO (25/80, 32.5%) [17, 30, 31, 34, 35, 44, 46, 51, 52, 55, 59, 60, 63, 64, 66, 69, 73, 75, 79, 84, 86, 89, 92–94]. Indoor environmental CO measurement was reported in 22 papers [33, 36, 37, 42, 44, 48, 50, 53, 54, 56, 65, 67, 70, 73, 82, 90, 95–100]. Whilst CO breath analysis was reported in 10 [38, 40, 52–54, 58, 61, 85, 86, 97]. Over half (54/80, 67.5%) used a single method of measurement [11, 16, 17, 24–32, 34, 36–39, 41–43, 45–50, 55–57, 62, 65–69, 71, 72, 74–78, 80, 81, 83, 84, 87–91, 95, 99, 100], while 26 (32.5%) reported 2 methods. [33, 35, 40, 44, 51–54, 58–61, 63, 64, 70, 73, 79, 82, 85, 86, 92–94, 96–98].

Overall papers varied by measurement method used, geographical setting, the setting in which the measurement was taken, whether they differentiated between smokers and non-smokers and research methodology. Three clinical guidelines were for use in both primary and secondary care [27, 28, 30], two in the emergency department [24, 29], one each in primary [26] and secondary care [16] and two set air quality standards [11, 25] (Table 2).

**Table 2:** Analysis method for all papers. Methodology, geographical setting, and setting of CO measurement for research studies.

	*Total papers *	*COHb (blood) *	COHb (oximetry)	*Breath analysis *	*Indoor CO levels *
**All papers (n = 80) **	80	48	25	10	22
*Smoking status undifferentiated *	28	15	9	2	10
*Smoking levels differentiated *	26	18	8	4	3
*No smoking levels reported *	26	15	8	4	9
**Research studies (n = 71) **	71	40	24	10	22
*Undifferentiated *	28	17	9	2	10
*Differentiate *	18	21	7	4	3
*No levels reported *	25	2	8	4	9
**Guidelines **	9	8	1	0	0
*Undifferentiated *	0	0	0	-	-
*Differentiate *	8	7	1	-	-
*No levels reported *	1	1	0	-	-
**Research papers (n = 71) **	*Total papers *	*COHb (blood) *	COHb (oximetry)	*Breath analysis *	*Indoor CO levels *
**Study type**					
*Cross-sectional*	38	19	14	7	12
*Case report and case series*	17	14	4	0	1
*Diagnostic accuracy*	3	3	3	0	0
*Cohort*	7	3	3	2	2
*Interventional *	6	1	0	1	7
**Study geographical setting**					
*Africa *	7	1	3	0	5
*Asia *	5	1	1	1	3
*Europe *	22	15	8	2	3
*North America *	25	17	9	5	7
*South America *	5	0	2	2	3
*Middle East *	4	4	1	0	0
*Southeast Asia *	2	2	0	0	0
*Mixed *	1	0	0	0	1
**Study setting (location of measurement)**					
*Emergency Department (ED) *	36	32	12	4	2
*Residence *	16	0	2	3	14
*Workplace *	5	1	2	0	4
*Recreation venue *	2	1	0	1	2
*P* *re-hospital*	1	0	1	0	0
*Hospital ward or outpatient clinic *	9	5	5	1	0
*Primary care*	1	1	0	0	0
*Not recorded *	1	0	1	0	0

### Diagnostic level used to determine CO exposure

Across all papers there were two units of measurement described. Parts per million were used to describe concentration of CO in the environment and COHb to describe levels of CO in participants’ blood. Regardless of method of measurement (blood, CO oximetry, expired or environmental) the diagnostic levels used to determine CO exposure were not stated in 32.5% (26) of papers [27, 28, 33, 36, 43, 49, 52, 53, 55, 66, 67, 69–73, 76, 80, 81, 83, 85, 86, 88, 92, 95, 98]. Of the 54 papers remaining 26 (32.5%) differentiated between CO levels in smokers and non-smokers [11, 16, 24, 26–30, 34, 40, 41, 46, 47, 51, 54, 59, 61, 68, 74, 77, 82, 84, 87, 89, 94, 97] and 28 (35.0%) did not [17, 31, 32, 35, 36, 38, 39, 42, 44, 45, 48, 50, 56–58, 60, 62–65, 75, 78, 79, 90, 91, 93, 96, 100].

**Fig 2:**
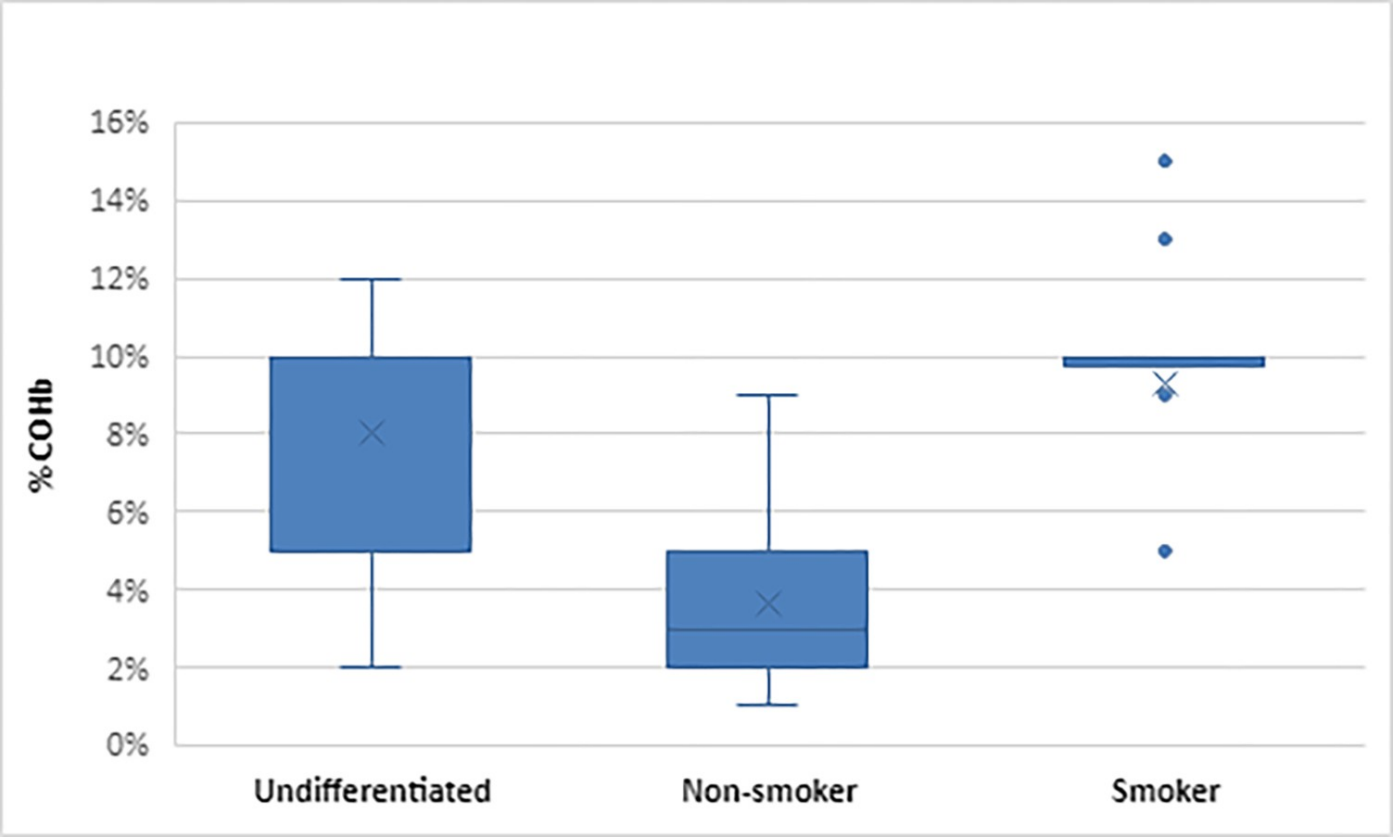
Box plot of COHb diagnostic levels reported in all papers (studies and guidelines).

**Fig 3:**
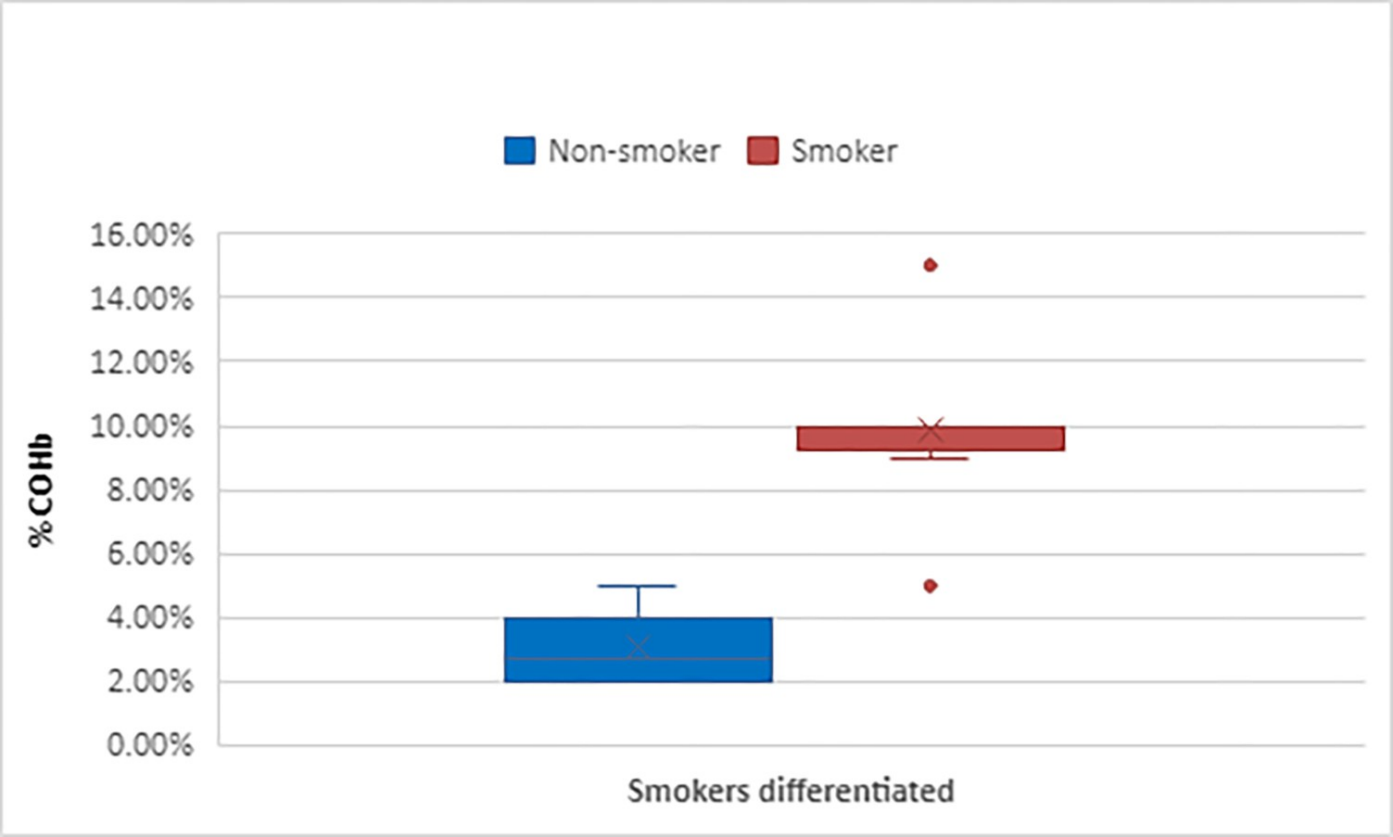
Box plot of COHb diagnostic levels reported in guidelines.

In papers reporting COHb levels, using either blood or CO oximetry measurement, (42,52.5%) [11, 16, 17, 24, 26–32, 34, 35, 39, 41, 44–47, 51, 57–64, 68, 74, 75, 77–79, 82, 84, 87, 89, 91, 93, 94, 96] the most frequently used level was reported as >/ = 10% (24/80; 30.0%) [11, 16, 17, 26, 29–32, 39, 41, 45, 51, 61, 63, 64, 74, 75, 77, 79, 82, 84, 87, 89, 94]. Papers differentiating between smoker and non-smokers (24,30.0%) [11, 16, 24, 26–30, 34, 40, 41, 46, 47, 51, 59, 61, 68, 74, 77, 82, 84, 87, 89, 94], the lowest level of COHb in non-smokers was >1% [77] the highest reported was a COHb >9% [59]. In smokers the lowest value given was COHb >5% [24, 34, 46, 68] and the highest COHb >15% [28]. In papers where smokers and non-smokers were not differentiated 22.5% (18/80) [17, 31, 32, 35, 39, 45, 57, 58, 60, 62–64, 75, 78, 79, 91, 93, 96] the lowest diagnostic level reported was 2% [62] and the highest 12% [60] (Figs 2 and 3). None of the studies reported the time between exposure and COHb measurement.

Fourteen papers reported in ppm using either ambient monitoring, [37, 42, 44, 48, 50, 56, 65, 90, 100] breath analysis [38, 58, 61] or both [54, 97]. Levels were wither reported as a discrete value at a single time point or as a level present for x number of hours.

#### Diagnostic threshold levels using blood COHb measurement

Blood COHb was reported in 48 papers [11, 16, 24–30, 32, 33, 35, 39–41, 43, 45, 47, 49, 51, 57, 58, 60–64, 68, 70–72, 74, 76–83, 85, 87, 88, 91–94, 96] of which 33 reported threshold levels [11, 16, 24, 26–29, 32, 35, 39–41, 45, 47, 51, 57, 58, 60–64, 68, 74, 77–79, 82, 87, 91, 93, 94, 96], 15 of which, did not differentiate between levels in smokers and non-smokers [32, 35, 39, 45, 57, 58, 60, 62–64, 78, 79, 91, 93, 96], with threshold levels between 2% [62] and 12% [60] in these papers.

Where papers reported values of both smokers and non-smokers (n = 18) [11, 16, 24, 26, 27, 29, 40, 41, 47, 51, 61, 68, 70, 74, 77, 82, 87, 94] these ranged from >5% [24, 58, 68] to >15% [28] in smokers and >1% [77] to >5% [24, 51, 74, 82, 94] in non-smokers.

#### SpCO diagnostic threshold levels

SpCO was used by 25 (31.3%) papers [17, 30, 31, 34, 35, 44, 46, 51, 52, 55, 59, 60, 63, 64, 66, 69, 73, 75, 79, 84, 86, 89, 92–94] of which 17 reported SpCO diagnostic threshold levels [17, 30, 31, 34, 35, 46, 51, 59, 60, 63, 64, 75, 79, 84, 89, 93, 94]. Nine papers did not differentiate between smokers and non-smokers [17, 31, 35, 60, 63, 64, 75, 79, 93] and used values of >4% [35] to >12% [60]. In the 8 studies differentiating smoking status [30, 34, 46, 51, 59, 84, 89, 94], non-smoker levels ranged from >2% [46] to >9% [59] and smoker values ranged from >5% [34, 46] to >13% [59]. The single guideline recommending SpCO measurement gave a diagnostic threshold of >4% for non-smokers and > 10% for smokers [30].

In the review agreement between SpCO and blood COHb was compared in 6 studies using Bland–Altman analysis, five of which reported small mean differences and clinically acceptable limits of agreement [51, 59, 60, 92–94], with a single paper reporting clinically unacceptable limits of agreement [60]. There was, however, a tendency for CO-oximetry to overestimate the SpCO relative to the COHb reported in all 6 studies [59, 93, 94]. A single paper suggested that the overestimate was due to the SpCO measurement being taken an average of 67 minutes before the blood COHb given the short half-life of COHb [59].

#### Breath analysis

Studies using end-tidal CO measurement breath analysers reported diagnostic threshold levels in 7 [38, 40, 52, 54, 58, 61, 97] of 10 papers [38, 40, 52–54, 58, 61, 85, 86, 97]. Smoking status was not differentiated in two studies that used diagnostic threshold levels of 6ppm and 9ppm [38, 58] respectively. One study converted ppm to COHb% and reported diagnostic threshold levels as 2% and 5% for non-smokers and smokers [40]. The remaining 3 studies that differentiated reported in ppm with values for non-smokers of 5.4, 6.91 and 15ppm and for smokers 16.2, 17 and 48ppm [54, 61, 97]. Breath analysis correlated well with blood COHb when measured contemporaneously, reporting r values > 0.7 in both children [58] adults [61] presenting to the ED. A single study measuring both SpCO and breath CO reported a high correlation; r = 0.79 [52]. In contrast, correlation between breath analysis and ambient monitoring was reported as poor (r = 0.17) [53].

#### Ambient CO measurement

22 studies measured ambient CO levels [28, 33, 36, 37, 42, 44, 48, 50, 53, 54, 56, 65, 67, 70, 73, 82, 90, 95–98, 100] of which 12 gave diagnostic cut off levels. The WHO guidance [11] was used in 7 studies [44, 54, 56, 90, 96, 97, 100] whilst 5 papers gave ranges from > 1.0ppm to > 9.4 ppm [37, 42, 48, 50, 65].

## Discussion

This scoping review presents a summary of the available research and clinical practice guidance of the current methods and diagnostic levels used to determine unintentional, non-fire-related CO exposure. We chose to carry out a scoping review to ensure a broad range of both research and clinical guidance was captured. We found four methods used to detect abnormal levels of CO (blood COHb, CO oximetry, breath analysis and ambient monitoring) but with variation in the stipulated levels used to determine exposure. We identified two principal areas for discussion, first the levels used to diagnose CO exposure and second, the method of CO measurement employed. The levels used in determining exposure varied widely across papers. This was true for papers using both COHb and CO concentration in ppm. The greatest variation was shown across research studies. Those reporting COHb ranged from 2–12% for undifferentiated studies, 1–9% for non-smokers, and 5–13% for smokers. Studies reporting ppm ranged from 1-25ppm for undifferentiated, 5.4-15ppm for non-smokers, 16.2-48ppm for smokers while clinical guidance ranging from 2.5–5% for non-smokers and 5–15% for smokers. This disparity in diagnostic threshold levels leads to variation in the diagnosis rates for CO exposure, either under-diagnosis or over-diagnosis depending on where the level is set. Whilst lower cut-off points may lead to over-diagnosis, this presents no impact on patient safety but may lead to an overutilisation of healthcare resources in the treatment of CO exposure. Using a higher threshold for determining if exposure has occurred could lead to under-diagnosis and the risk that the patient may be falsely reassured and discharged back to an environment where they are continually exposed to low levels of CO. Ongoing CO exposure can lead to long term health effects including delayed neurological sequalae and neuropsychological issues [104]. Guidelines were focused on diagnosis and treatment of patients with known CO exposure, with the implicit assumption that the clinician had already determined CO as a cause for the patient’s symptoms. The difficulty in diagnosing occult, low-level exposure is well documented as clinicians frequently mistake the vague symptoms for another cause [104–107]. We found no evidence of how levels of CO in ongoing exposure might differ from levels in acute exposure or be detected through the commonly used methods of screening in healthcare setting (blood or breath analysis and CO oximetry). Variance in levels used to determine exposure to CO in research papers has the potential to lead to heterogeneity in reporting of prevalence and lack of comparable data across studies. The predominant method reported for CO measurement was blood COHb. COHb is known to have deficiencies in its’ utility due to its short half-life of 137 to 240 minutes [108, 109] which is further reduced to 74 minutes when treated with high-flow oxygen at atmospheric pressure [110]. Patients may have levels of COHb that do not exceed the diagnostic threshold when measured if sufficient time has passed since exposure and this becomes more likely if oxygen is administered [110]. It is known that COHb levels correlate poorly with the patients’ clinical picture and degree of severity of exposure [111]. Despite these deficiencies blood COHb is still widely used in the hospital setting and was the recommended analytical method in eight of the nine clinical guideline documents. No studies reported the time between exposure and COHb measurement. This may lead to under-diagnosis of CO exposure in clinical practice as COHb may not be raised when measured at the time of clinical consultation. CO-oximetry was the second most common method used to measure CO levels. All studies used the Masimo Rad-57® Pulse CO-Oximeter®, a handheld battery-operated portable device that does not require regular calibration [112]. The majority of studies comparing CO-oximetry with blood COHb, Bland-Altman analysis reported small mean differences and moderate limits of agreement but with a tendency to overestimate the SpCO relative to the COHb reported. A recently published systematic review concluded that SpCO was “probably” not accurate enough to either confirm or exclude a diagnosis of CO exposure with certainty [113 (P233)]. However, the findings were limited by conducting a Bland Altman analysis of the complete data set. Despite these limitations, SpCO can be deployed in settings where blood COHb is not available such as in primary and prehospital care. CO breath analysis correlated well with blood COHb and SpCO but poorly with ambient monitoring. The utility of CO breath analysis in clinical practice is reduced by both device and individual patient factors. Devices require a breath hold of 15–20 seconds before measurement to ensure equilibrium between alveolar CO and COHb [114]. This can be challenging for patients with dyspnoea [115]. One of the clinical guidelines recommend its use. Ambient CO levels were recorded using either personal ‘wearable’ or static environmental monitoring devices. In research it has been used to confirm the source of CO. Harmful CO exposure depends both on CO level and duration of exposure. Safe exposure limits are defined in the WHO and the National Research Council guidance [11, 25]. Ambient CO detection in isolation is not enough to detect harmful exposure, and forms one part of the triad for diagnosis [16]. For patients presenting with symptoms that could be related to CO exposure but in whom COHb is normal ambient monitoring may provide an indication of the cause of symptoms as it overcomes the limitation of delayed COHb measurement by measuring CO levels at source. Currently this analytical method is not readily available to clinicians in practice and is only used in research.

## Strengths and limitations

Our review followed the methodology described by Arksey and O’Malley and included both online databases and the grey literature. The reference lists of all included papers were hand searched to ensure as wide a coverage of the topic area as possible. The review does have some limitations. We did not undertake a quality appraisal of the literature in the review which could have introduced bias into our findings. The search was current up to August 2023 and therefore we may have missed subsequent relevant papers published of in press. Finally, we chose to exclude papers written in languages other than English which also may have missed relevant papers.

## Conclusion

This review included 80 papers discussing the diagnostic methods for detecting unintentional low-level (UNFR) CO exposure. Findings suggest there is limited international agreement on the CO levels used to determine exposure in research papers. Variation was more heterogeneous in clinical guidelines. Clinical diagnosis of UNFR CO exposure is important to reduce the effects of both acute and chronic exposure. Diagnostic measures for determining CO exposure should be used as part of the triad of 1. symptoms consistent with CO exposure; 2. a recent history of CO exposure; and 3. elevated levels of COHb. Defining what constitutes CO exposure is difficult due to the variability of levels presented in papers which may lead to over- or under-diagnosis depending on the level used. Variation leads to difficulty in determining the true prevalence of UNFR globally. We recommend that researchers consider standardising the values used to determine abnormally high CO to allow for comparability of results and enable an accurate picture of CO exposure worldwide.

## Supporting information

S1 Checklist PRISMA SCR checklist. (DOCX)

S1 Table Search strategy. (DOCX)

S2 Table Data extraction variables. (DOCX)

S3 Table a. Characteristics of papers. b. Analysis methods and diagnostic levels.
(ZIP)

S1 File (XLSX)

S2 File (XLSX)
