# Supplementary material for: Diagnosis of carbon monoxide exposure in clinical research and practice: A scoping review
Source: PLoS One. 2025 Feb 5;20(2):e0300989. doi: 10.1371/journal.pone.0300989 (PMC11798492; doi:10.1371/journal.pone.0300989)
Supplement: S1 Table — (DOCX) [file pone.0300989.s002.docx]

**S1 Table**

**Search strategy**

| Database | Search strategy | Number of results |
| --- | --- | --- |
| Embase (Ovid)  1974 to 2022 March 07      Date searched: 8/3/2022 | 1(carbon monoxide and (expos* or intoxicat* or poisoned or poisoning*)).tw.  2carbon monoxide intoxication/  31 or 2  4(domestic or home or homes or house* or indoor* or occupational or residence* or residential or work or workplace*).tw.  5Housing/  6indoor air pollution/  7or/4-6  8(diagnos* or determin* or identif* or recogni*).tw.  9exp Diagnosis/  10diagnosis.fs.  11or/8-10  123 and 7 and 11  13exp animal/ not exp human/  14(cigarette* or hookah or shisha* or tobacco or waterpipe*).tw,kf.  1512 not (13 or 14)  16limit 15 to (english language and yr="2002 -Current") | 740 |
| Medline (Ovid)  1946 to March 07, 2022      Date searched: 8/3/2022 | 1(carbon monoxide and (expos* or intoxicat* or poisoned or poisoning*)).tw.  2Carbon Monoxide Poisoning/  31 or 2  4(domestic or home or homes or house* or indoor* or occupational or residence* or residential or work or workplace*).tw.  5Housing/  6Air Pollution, Indoor/  7or/4-6  8(diagnos* or determin* or identif* or recogni*).tw.  9exp Diagnosis/  10diagnosis.fs.  11or/8-10  123 and 7 and 11  13exp animals/ not exp humans/  14(cigarette* or hookah or shisha* or tobacco or waterpipe*).tw,kf.  1512 not (13 or 14)  16limit 15 to (english language and yr="2002 -Current") | 529 |
| Cinahl (EBSCO)        Date searched: 8/3/2022 | 1 TI (carbon monoxide and (expos* or intoxicat* or poisoned or poisoning*)) or AB (carbon monoxide and (expos* or intoxicat* or poisoned or poisoning*))  2 (MH "Carbon Monoxide Poisoning")  3 1 or 2  4 TI (domestic or home or homes or house* or indoor* or occupational or residence* or residential or work or workplace*) or AB (domestic or home or homes or house* or indoor* or occupational or residence* or residential or work or workplace*)  5 (MH "Housing")  6 (MH "Air Pollution, Indoor")  7 4 or 5 or 6  8 TI (diagnos* or determin* or identif* or recogni*) or AB (diagnos* or determin* or identif* or recogni*)  9 (MH "Diagnosis+")  10 MW "DI"  11 8 or 9 or 10  12 (MH "Animals+") not (MH "Humans+")  13 TI (cigarette* or hookah or shisha* or tobacco or waterpipe*) or AB (cigarette* or hookah or shisha* or tobacco or waterpipe*)  14 3 and 7 and 11  15 14 not (12 or 13) | 221 |
