# Supplementary material for: Diagnosis of carbon monoxide exposure in clinical research and practice: A scoping review
Source: PLoS One. 2025 Feb 5;20(2):e0300989. doi: 10.1371/journal.pone.0300989 (PMC11798492; doi:10.1371/journal.pone.0300989)
Supplement: S2 Table — (DOCX) [file pone.0300989.s003.docx]

**S2 Table**

**Data extraction variables**

| Title |  |
| --- | --- |
| First author |  |
| Article type | - Original research - Case study - Conference proceedings - Clinical Guideline |
| Year |  |
| Aim |  |
| Region | - Africa - Asia - Australasia - Europe - Middle East - North Africa - North America - South America - Southeast Asia - International |
| Country |  |
| Study design | - Case report - Case series - Cohort - Cross sectional - Interventional - Diagnostic accuracy - Guidelines |
| Study setting (where measurement taken) | - Ambulance - Emergency Department - Environmental (home, workplace, leisure, mixed) - Hospital - Guideline |
| Population | - Adult - Child - Adult and child |
| Sample size | N = |
| Co Exposure | - Known source - Not known source |
| Source of exposure setting | - Domestic - Occupational - Recreational - Mixed |
| Type of CO level measurement | - COHb (venous blood) - SpCO - Ambient CO monitor - Breath analyser |
| CO exposure diagnostic threshold level | - Smoker and non-smoker combined - Smoker - Non-smoker |
| CO level reported | - ppm - % COHb |
| Time from exposure to CO level measurement | - minutes |
