# Supplementary material for: Diagnosis of carbon monoxide exposure in clinical research and practice: A scoping review
Source: PLoS One. 2025 Feb 5;20(2):e0300989. doi: 10.1371/journal.pone.0300989 (PMC11798492; doi:10.1371/journal.pone.0300989)
Supplement: S3 Table — a. Characteristics of papers. b. Analysis methods and diagnostic levels. (ZIP) [file pone.0300989.s004.zip › S3b_Table.docx]

**S3b Table**

**All papers (studies and guidelines) measurement source and diagnostic levels**

| **Author year (country)** | ***Ref number*** | **Diagnostic levels reported?**  **(Yes/No)** | **Source of measurement 1** | **Overall diagnostic** **level**  **1** | **Non-smoker diagnostic level**  **1** | **Smoker diagnostic level**  **1** | **Source of measurement2** | **Overall diagnostic level**  **2** | **Non-smoker diagnostic level**  **2** | **Smoker diagnostic level**  **2** |
| --- | --- | --- | --- | --- | --- | --- | --- | --- | --- | --- |
| **Studies** | | | | | | | | | | |
| Ablesohn et al 2002 (Canada) | 68 | Yes | COHb (blood) | Not applicable | 3% | 5% | Not applicable | Not applicable | Not applicable | Not applicable |
| Acharya 2021 (Nepal) | 31 | Yes | SpCO (oximeter) | 10% | Not applicable | Not applicable | Not applicable | Not applicable | Not applicable | Not applicable |
| Ahmed et al 2015 (USA) | 69 | No | SpCO (oximeter) | No levels reported | No levels reported | No levels reported | Not applicable | Not applicable | Not applicable | Not applicable |
| Aksu et al 2012 (Turkey) | 32 | Yes | COHb (blood) | 10% | Not applicable | Not applicable | Not applicable | Not applicable | Not applicable | Not applicable |
| Alexander et al 2013 (Bolivia) | 99 | No | Ambient CO levels | No levels reported | No levels reported | No levels reported | Not applicable | Not applicable | Not applicable | Not applicable |
| Audin 2006 (USA) | 70 | No | COHb (blood) | No levels reported | No levels reported | No levels reported | Ambient CO levels | Not reported | Not reported | Not reported |
| Ayalloore et al 2012 (USA) | 71 | No | COHb (blood) | No levels reported | No levels reported | No levels reported | Not applicable | Not applicable | Not applicable | Not applicable |
| Balakrishnan et al 2015 (India) | 98 | No | Ambient CO levels | No levels reported | No levels reported | No levels reported | Ambient CO levels | Not reported | Not reported | Not reported |
| Balzan, Cacciottolo, & Mifsud 1994 (Malta) | 72 | No | COHb (blood) | No levels reported | No levels reported | No levels reported | Not applicable | Not applicable | Not applicable | Not applicable |
| Banjoko et al 2007 (Nigeria) | 33 | No | Ambient CO levels | No levels reported | No levels reported | No levels reported | COHb (blood) | Not reported | Not reported | Not reported |
| Barker et al 2006 (USA) | 92 | No | SpCO (oximeter) | No levels reported | No levels reported | No levels reported | COHb (blood) | Not reported | Not reported | Not reported |
| Bledsoe et al 2010 (USA) | 73 | No | Ambient CO levels | No levels reported | No levels reported | No levels reported | SpCO (oximeter) | Not reported | Not reported | Not reported |
| Bol et al 2018 (Turkey) | 84 | Yes | SpCO (oximeter) | Not applicable | 5% | 10% | Not applicable | Not applicable | Not applicable | Not applicable |
| Chan 2017 (Hong Kong) | 74 | Yes | COHb (blood) | Not applicable | 5% | 10% | Not applicable | Not applicable | Not applicable | Not applicable |
| Chee et al 2008 (USA) | 75 | Yes | SpCO (oximeter) | 10% | Not applicable | Not applicable | Not applicable | Not applicable | Not applicable | Not applicable |
| Clarke et al 2012 (UK) | 34 | Yes | SpCO (oximeter) | Not applicable | 2.5% | 5% | Not applicable | Not applicable | Not applicable | Not applicable |
| Cooper et al 2020 (USA) | 35 | Yes | SpCO (oximeter) | 4% | Not applicable | Not applicable | COHb (blood) | 4% | Not applicable | Not applicable |
| Creswell et al 2015 (USA) | 85 | No | COHb (blood) | No levels reported | No levels reported | No levels reported | CO Breath analyser | Not reported | Not reported | Not reported |
| Croxford et al 2005 (UK) | 37 | Yes | Ambient CO levels | 8.6ppm | Not applicable | Not applicable | Not applicable | Not applicable | Not applicable | Not applicable |
| Croxford et al 2005 (UK) | 36 | No | Ambient CO levels | No levels reported | No levels reported | No levels reported | Not applicable | Not applicable | Not applicable | Not applicable |
| Cunnington & Hormbrey 2002 (UK) | 38 | Yes | CO breath analyzer | 6ppm | Not applicable | Not applicable | Not applicable | Not applicable | Not applicable | Not applicable |
| Deniz et al 2017 (Turkey) | 17 | Yes | SpCO (oximeter) | 10% | Not applicable | Not applicable | Not applicable | Not applicable | Not applicable | Not applicable |
| Dolan et al 1987 (USA) | 39 | Yes | COHb (blood) | 10% | Not applicable | Not applicable | Not applicable | Not applicable | Not applicable | Not applicable |
| Eberhardt et al 2006 (USA) | 40 | Yes | CO breath analyzer | Not applicable | 2% | 5% | COHb (blood) | Not applicable | 2% | 5% |
| El Sayed & Tamim 2014 (Lebanon) | 41 | Yes | COHb (blood) | Not applicable | 3% | 10% | Not applicable | Not applicable | Not applicable | Not applicable |
| Fandino-Del-Rio et al 2020 (Peru) | 42 | Yes | Ambient CO levels | 9.4 ppm | Not applicable | Not applicable | Not applicable | Not applicable | Not applicable | Not applicable |
| Guven & Sarici 2023 (Turkey) | 91 | Yes | COHb (blood) | 5% | Not applicable | Not applicable | Not applicable | Not applicable | Not applicable | Not applicable |
| Hampson & Dunn 2015 (USA) | 43 | No | COHb (blood) | No levels reported | No levels reported | No levels reported | Not applicable | Not applicable | Not applicable | Not applicable |
| Havens et al 2018 (Malawi) | 44 | Yes | Ambient CO levels | WHO ppm recommendations | Not applicable | Not applicable | SpCO (oximeter) | Not reported | Not reported | Not reported |
| Heckerling 1987 (USA) | 45 | Yes | COHb (blood) | 10% | Not applicable | Not applicable | Not applicable | Not applicable | Not applicable | Not applicable |
| Hol et al 2012 (Netherlands) | 76 | No | COHb (blood) | No levels reported | No levels reported | No levels reported | Not applicable | Not applicable | Not applicable | Not applicable |
| Hubbell et al 2013 (Haiti) | 46 | Yes | SpCO (oximeter) | Not applicable | 2% | 5% | Not applicable | Not applicable | Not applicable | Not applicable |
| Hullin et al 2017 (France) | 86 | No | SpCO (oximeter) | No levels reported | No levels reported | No levels reported | CO Breath analyser | Not reported | Not reported | Not reported |
| Jarman et al 2023 (UK) | 47 | Yes | COHb (blood) | Not applicable | 2% | 6% | Not applicable | Not applicable | Not applicable | Not applicable |
| Jaslow et al 2001 (USA) | 48 | Yes | Ambient CO levels | 1.0 ppm | Not applicable | Not applicable | Not applicable | Not applicable | Not applicable | Not applicable |
| Johnson et al 2022 (Guatemala, India, Peru, & Rwanda) | 100 | Yes | Ambient CO levels | 3.5ppm in 24 hours (WHO Guidance) | Not applicable | Not applicable | Not applicable | Not applicable | Not applicable | Not applicable |
| Keles et al 2008 (Turkey) | 49 | No | COHb (blood) | No levels reported | No levels reported | No levels reported | Not applicable | Not applicable | Not applicable | Not applicable |
| Kirkham et al 2011 (Canada) | 50 | Yes | Ambient CO levels | 25ppm | Not applicable | Not applicable | Not applicable | Not applicable | Not applicable | Not applicable |
| Koyuncu et al 2020 (Turkey) | 51 | Yes | SpCO (oximeter) | Not applicable | 5% | 10% | COHb (blood) | Not applicable | 5% | 10% |
| Lam et al 2020 (Guatemala) | 52 | No | CO breath analyzer | No levels reported | No levels reported | No levels reported | SpCO (oximeter) | Not reported | Not reported | Not reported |
| Lee et al 2015 (Bangladesh) | 53 | No | Ambient CO levels | No levels reported | No levels reported | No levels reported | CO Breath analyser | Not reported | Not reported | Not reported |
| Levesque et al 2005 (Canada) | 54 | Yes | CO breath analyzer | Not applicable | 5.4ppm | 16.2ppm | Ambient CO levels | 25 ppm for 60 minutes | Not reported | Not reported |
| Liu et al 2022 (China) | 87 | Yes | COHb (blood) | Not applicable | 3% | 10% | Not applicable | Not applicable | Not applicable | Not applicable |
| McGuiffe et al 2000 et al (UK) | 77 | Yes | COHb (blood) | Not applicable | 1% | 10% | Not applicable | Not applicable | Not applicable | Not applicable |
| Medhane 2018 (USA) | 78 | Yes | COHb (blood) | 5% | Not applicable | Not applicable | Not applicable | Not applicable | Not applicable | Not applicable |
| Mortlemans et al 2013 (Belgium) | 88 | No | COHb (blood) | No levels reported | No levels reported | No levels reported | Not applicable | Not applicable | Not applicable | Not applicable |
| Nilson et al 2010 (USA) | 55 | No | SpCO (oximeter) | No levels reported | No levels reported | No levels reported | Not applicable | Not applicable | Not applicable | Not applicable |
| North et al 2019 (Uganda) | 90 | Yes | Ambient CO levels | 3.5ppm in 24 hours | Not applicable | Not applicable | Not applicable | Not applicable | Not applicable | Not applicable |
| Pope et al 2015 (Guatemala) | 97 | Yes | CO breath analyzer | Not applicable | 6.91 PPM | 17 PPM | Ambient CO levels | 6 ppm for 24 hours | Not reported | Not reported |
| Rabbani et al 2022 (Pakistan) | 65 | Yes | Ambient CO levels | 1.00ppm | Not applicable | Not applicable | Not applicable | Not applicable | Not applicable | Not applicable |
| Roth et al 2011 (Austria) | 93 | Yes | COHb (blood) | 10% | Not applicable | Not applicable | SpCO (oximeter) | 10% | Not applicable | Not applicable |
| Roth et al 2013 (Austria) | 80 | No | COHb (blood) | No levels reported | No levels reported | No levels reported | Not applicable | Not applicable | Not applicable | Not applicable |
| Roth et al 2011 (Austria) | 79 | Yes | SpCO (oximeter) | 6.60% | Not applicable | Not applicable | COHb (blood) | 6.60% | Not applicable | Not applicable |
| Rylance et al 2019 (Malawi) | 56 | Yes | Ambient CO levels | WHO 81ppm for 15 mins exposure | Not applicable | Not applicable | Not applicable | Not applicable | Not applicable | Not applicable |
| Salameh et al 2009 (Israel) | 57 | Yes | COHb (blood) | 5% | Not applicable | Not applicable | Not applicable | Not applicable | Not applicable | Not applicable |
| Sebbane et al 2013 (France) | 94 | Yes | SpCO (oximeter) | Not applicable | 5% | 10% | COHb (blood) | Not applicable | 5% | 10% |
| Shenoi et al 1998 (USA) | 58 | Yes | CO breath analyzer | 9 ppm | Not applicable | Not applicable | COHb (blood) | 5% | Not applicable | Not applicable |
| Silver et al 2023 (USA) | 67 | No | Ambient CO levels | No levels reported | No levels reported | No levels reported | Not applicable | Not applicable | Not applicable | Not applicable |
| Suliman et al 2021 (Sudan) | 89 | Yes | SpCO (oximeter) | Not applicable | 5% | 10% | Not applicable | Not applicable | Not applicable | Not applicable |
| Suner et al 2008 (USA) | 59 | Yes | SpCO (oximeter) | Not applicable | 9% | 13% | COHb (blood) | Not reported | Not reported | Not reported |
| Tetsuku et al 2021 (Japan) | 81 | No | COHb (blood) | No levels reported | No levels reported | No levels reported | Not applicable | Not applicable | Not applicable | Not applicable |
| Thomassen et al 2004 (Norway) | 96 | Yes | COHb (blood) | 5% | Not applicable | Not applicable | Ambient CO levels | 35 ppm for 8 hours | Not applicable | Not applicable |
| Touger et al 2010 (USA) | 60 | Yes | SpCO (oximeter) | 12% | Not applicable | Not applicable | COHb (blood) | 12% | Not applicable | Not applicable |
| Turhan et al 2014 (Turkey) | 83 | No | COHb (blood) | No levels reported | No levels reported | No levels reported | Not applicable | Not applicable | Not applicable | Not applicable |
| Turnbull et al 1988 (USA) | 61 | Yes | CO breath analyzer | Not applicable | 15 ppm | 48ppm | COHb (blood) | Not applicable | 3% | 10% |
| Unsal et al 2015 (Turkey) | 62 | Yes | COHb (blood) | 2% | Not applicable | Not applicable | Not applicable | Not applicable | Not applicable | Not applicable |
| Villalba et al 2019 (USA) | 63 | Yes | SpCO (oximeter) | 10% | Not applicable | Not applicable | COHb (blood) | 10% | Not applicable | Not applicable |
| Wilson et al 2018 (USA) | 82 | Yes | COHb (blood) | Not applicable | 5% | 10% | Ambient CO levels | Not reported | Not reported | Not reported |
| Yip et al 2017 (Kenya) | 95 | No | Ambient CO levels | No levels reported | No levels reported | No levels reported | Not applicable | Not applicable | Not applicable | Not applicable |
| Zoller et al 2022 (Tanzania) | 66 | No | SpCO (oximeter) | No levels reported | No levels reported | No levels reported | Not applicable | Not applicable | Not applicable | Not applicable |
| Zorbalar et al 2014 (Turkey) | 64 | Yes | SpCO (oximeter) | 10% | Not applicable | Not applicable | COHb (blood) | 10% | Not applicable | Not applicable |
| **Guidelines** | | | | | | | | | | |
| National Research Council 2010 (USA) | 25 | No | COHb (blood) | No levels reported | No levels reported | No levels reported | Not applicable | Not applicable | Not applicable | Not applicable |
| WHO 2021 | 11 | Yes | COHb (blood) | Not applicable | 2.50% | 10% | Not applicable | Not applicable | Not applicable | Not applicable |
| BMJ 2016 (UK) | 28 | Yes | COHb (blood) | Not applicable | 2% | 15% | Not applicable | Not applicable | Not applicable | Not applicable |
| CDC 2020 (USA) | 27 | Yes | COHb (blood) | Not applicable | 2% | 9% | Not applicable | Not applicable | Not applicable | Not applicable |
| Juttner et al 2021 (Germany) | 30 | Yes | SpCO (oximeter) | Not applicable | 4% | 10% | Not applicable | Not applicable | Not applicable | Not applicable |
| NICE 2023 (UK) | 26 | Yes | COHb (blood) | Not applicable | 2% | 10% | Not applicable | Not applicable | Not applicable | Not applicable |
| Am. Coll. of Emergency Medicine 2016 (USA) | 29 | Yes | COHb (blood) | Not applicable | 3% | 10% | Not applicable | Not applicable | Not applicable | Not applicable |
| Austin Toxicology 2021  (Australia) | 24 | Yes | COHb (blood) | 5% | Not applicable | Not applicable | Not applicable | Not applicable | Not applicable | Not applicable |
| Hampson et al 2012 (USA) | 16 | Yes | COHb (blood) | Not applicable | 5% | 10% | Not applicable | Not applicable | Not applicable | Not applicable |
