# Supplementary material for: Diagnosis of carbon monoxide exposure in clinical research and practice: A scoping review
Source: PLoS One. 2025 Feb 5;20(2):e0300989. doi: 10.1371/journal.pone.0300989 (PMC11798492; doi:10.1371/journal.pone.0300989)
Supplement: S3 Table — a. Characteristics of papers. b. Analysis methods and diagnostic levels. (ZIP) [file pone.0300989.s004.zip › S3a_Table.docx]

**S3a Table**

**Study characteristics**

| **Author year**  **(country)** | ***Reference number*** | **Study design** | **Study location** | **Population**  **(Adult / Children / Both)** | **Sample size** | **Known exposure to CO** **(Yes /No)** | **Place of exposure** |
| --- | --- | --- | --- | --- | --- | --- | --- |
| Ablesohn et al 2002 (Canada) | 68 | Case report | Not reported | Children | 1 | No | Domestic |
| Acharya 2021  (Nepal) | 31 | Cross sectional | Hospital | Adult | 1040 | No | Domestic |
| Ahmed et al 2015  (USA) | 69 | Case report | Hospital | Adult | 1 | Yes | Domestic |
| Aksu et al 2012  (Turkey) | 32 | Cross sectional | Emergency Department | Adult | 476 | Yes | Not known |
| Alexander et al 2013 (Bolivia) | 99 | Interventional | Environmental (home) | Adult | 20 | No | Domestic |
| Audin 2006  (USA) | 70 | Case report | Emergency Department | Both | 16 | Yes | Domestic |
| Ayalloore et al 2012 (USA) | 71 | Case report | Emergency Department | Adult | 1 | Yes | Occupational |
| Balakrishnan et al 2015 (India) | 98 | Interventional | Environmental (home) | Adult | 65 | Yes | Domestic |
| Balzan et al 1994 (Malta) | 72 | Case report | Emergency Department | Adult | 104 | No | Various |
| Banjoko et al 2007 (Nigeria) | 33 | Cross sectional | Environmental (occupational) | Adult | 100 | No | Occupational |
| Barker et al 2006 (USA) | 92 | Diagnostic accuracy | Hospital | Adult | 20 | Yes | Experimental inhalation |
| Bledsoe et al 2010 (USA) | 73 | Case report | Environmental (occupational) | Adult | Not reported | Yes | Occupational |
| Bol et al 2018  (Turkey) | 84 | Cohort | Environmental (occupational) | Adult | 99 | Yes | Vehicle |
| Chan 2017  (Hong Kong) | 74 | Case report | Hospital | Adult | 1 | No | Domestic |
| Chee et al 2008 (USA) | 75 | Case series | Emergency Department | Adult | 74880 | No | Various |
| Clarke et al 2012 (UK) | 34 | Cross sectional | Emergency Department | Adult | 1758 | No | Various |
| Cooper et al 2020 (USA) | 35 | Cross sectional | Emergency Department | Both | 4003 | No | Not known |
| Creswell et al 2015 (USA) | 85 | Cohort | Hospital | Adult | 92 | Yes | Recreational |
| Croxford et al 2005 (UK) | 37 | Cross sectional | Environmental (home) | Both | 270 | No | Domestic |
| Croxford et al 2005 (UK) | 36 | Cross sectional | Environmental (home) | Both | 56 | No | Domestic |
| Cunnington & Hormbrey 2002 (UK) | 38 | Cross sectional | Emergency Department | Adult | 366 | No | Not known |
| Deniz et al 2017 (Turkey) | 17 | Cross sectional | Emergency Department | Both | 1788 | No | Not known |
| Dolan et al 1987  (USA) | 39 | Cross sectional | Emergency Department | Adult | 55 | No | Domestic |
| Eberhardt et al 2006 (USA) | 40 | Cross sectional | Emergency Department | Adult | 170 | Yes | Various |
| El Sayed & Tamim 2014 (Lebanon) | 41 | Cross sectional | Emergency Department | adult | 27 | Yes | Various |
| Fandino-Del-Rio et al 2020 (Peru) | 42 | Cross sectional | Environmental (home) | Both | 180 | Yes | Domestic |
| Guven & Sarici 2023 (Turkey) | 91 | Cohort | Emergency Department | Children | 760 | Yes | Domestic |
| Hampson & Dunn 2015 (USA) | 43 | Cross sectional | Hospital | Both | 264 | Yes | Various |
| Havens et al 2018 (Malawi) | 44 | Cross sectional | Environmental (home) | Children | 1928 | Yes | Domestic |
| Heckerling 1987  (USA) | 45 | Cross sectional | Emergency Department | Adult | 37 | No | Domestic |
| Hol et al 2012 (Netherlands) | 76 | Case report | Emergency Department | Adult | 1 | No | Domestic |
| Hubbell et al 2013 (Haiti) | 46 | Cross sectional | Hospital | Both | 2296 | Yes | Domestic |
| Hullin et al 2017 (France) | 86 | Cohort | not reported | Both | 3028 | Yes | Various |
| Jarman et al 2023  (UK) | 47 | Cross sectional | Emergency Department | Adult | 4175 | No | Not known |
| Jaslow et al 2001  (USA) | 48 | Cross sectional | Environmental (home) | Not reported | 264 | No | Domestic |
| Johnson et al 2022 (Guatemala, India, Peru, & Rwanda) | 100 | Randomised control trial | Environmental (home) | Adult | 3195 | Yes | Domestic |
| Keles et al 2008  (Turkey) | 49 | Cross sectional | Emergency Department | Both | 323 | No | Domestic |
| Kirkham et al 2011 (Canada) | 50 | Cross sectional | Environmental (occupational) | Adult | 45 | Yes | Occupational |
| Koyuncu et al 2020 (Turkey) | 51 | Cross sectional | Emergency Department | Adult | 4073 | No | Domestic |
| Lam et al 2020 (Guatemala) | 52 | Cross sectional | Environmental (home) | Both | 66 | Yes | Domestic |
| Lee et al 2015 (Bangladesh) | 53 | Cross sectional | Environmental (home) | Adult | 28 | Yes | Domestic |
| Levesque et al 2005 (Canada) | 54 | Cross sectional | Environmental (recreational) | both | 10 | Yes | Recreational |
| Liu et al 2022  (China) | 87 | Cohort | Emergency Department | Adult | 314 | Yes | Domestic |
| McGuiffe et al 2000 (UK) | 77 | Case report | Emergency Department | Adult | 22 | Yes | Recreational |
| Medhane et al 2018  (USA) | 78 | Case report | Hospital | Adult | 2 | Yes | Domestic |
| Mortlemans et al 2013 (Belgium) | 88 | Cohort | Emergency Department | Both | 91 | Yes | Recreational |
| Nilson et al 2010  (USA) | 55 | Cross sectional | Ambulance service | Adult | 1093 | No | Not known |
| North et al 2019 (Uganda) | 90 | Cohort | Environmental (home) | Adult | 260 | Yes | Domestic |
| Pope et al 2015 (Guatemala) | 97 | Interventional | Environmental (home) | Adult | 504 | Yes | Domestic |
| Rabbani et al 2022 (Pakistan) | 65 | Cross sectional | Environmental (home) | Adult | 1629 | No | Not known |
| Roth et al 2011 (Austria) | 93 | Case report | Emergency Department | Adult | 1 | Yes | Domestic |
| Roth et al 2013 (Austria) | 80 | Case report | Emergency Department | Adult | 5 | Yes | Domestic |
| Roth et al 2011 (Austria) | 79 | Diagnostic accuracy | Emergency Department | Adult | 1578 | No | Not known |
| Rylance et al 2019 (Malawi) | 56 | Cross sectional | Environmental (home) | Children | 804 | Yes | Domestic |
| Salameh et al 2009 (Israel) | 57 | Cross sectional | Emergency Department | Both | 292 | Yes | Various |
| Sebbane et al 2013 (France) | 94 | Diagnostic accuracy | Emergency Department | Adult | 93 | No | Not known |
| Shenoi et al 1998  (USA) | 58 | Cross sectional | Emergency Department | Both | 470 | No | Not known |
| Silver et al 2023  (USA) | 67 | Cross sectional | Environmental (occupational) | Adult | 16 | Yes | Occupational |
| Suliman et al 2021 (Sudan) | 89 | Cohort | Environmental (home) | Both | 157 | Yes | Domestic |
| Suner et al 2008  (USA) | 59 | Cross sectional | Emergency Department | Adult | 14438 | No | Not known |
| Tetsuku et al 2021 (Japan) | 81 | Case report | Emergency Department | Adult | 1 | Yes | Domestic |
| Thomassen et al 2004 (Norway) | 96 | Interventional | Environmental (recreational) | Adult | 7 | Yes | Recreational |
| Touger et al 2010  (USA) | 60 | Cross sectional | Emergency Department | Both | 120 | Yes | Various |
| Turhan et al 2014 (Turkey) | 83 | Case report | Emergency Department | Adult | 1 | Yes | Domestic |
| Turnbull et al 1988 (USA) | 61 | Cross sectional | Emergency Department | Adult | 504 | No | Not known |
| Unsal et al 2015 (Turkey) | 62 | Cross sectional | Emergency Department | Children | 80 | Yes | Domestic |
| Villalba et al 2019  (USA) | 63 | Cross sectional | Emergency Department | Both | 126 | No | Not known |
| Wilson et al 2018  (USA) | 82 | Case series | Emergency Department | Adult | 37 | Yes | Occupational |
| Yip et al 2017  (Kenya) | 95 | Interventional | Environmental (home) | Both | 48 | Yes | Domestic |
| Zoller et al 2022 (Tanzania) | 66 | Cross sectional | Hospital | Adult | 997 | Yes | Domestic |
| Zorbalar et al 2014  (Turkey) | 64 | Cross sectional | Emergency Department | Both | 482 | Yes | Domestic |
